# The consequences of after-hours work: a fixed-effect study of burnout, pain, detachment and work–home conflict among Norwegian workers

**DOI:** 10.5271/sjweh.4198

**Published:** 2025-01-01

**Authors:** Vilde Hoff Bernstrøm, Mari Ingelsrud, Wendy Nilsen

**Affiliations:** 1 Work Research Institute, OsloMet – Oslo Metropolitan University, Oslo, Norway.

**Keywords:** extended work hour, extended daily work hour, longitudinal, quick return, working time

## Abstract

**Objectives:**

Working outside the workplace and ordinary work hours has become common for a larger part of the working population. The objective of the current study was to examine the relationship between working after-hours and employee burnout, musculoskeletal pain, detachment and work–home conflict, delineating the independent effect of four different types of after-hours work, and the moderating role of work-time control.

**Methods:**

The data comprised longitudinal questionnaire data from 1465 full-time employees in Norway across four waves (2021–2022). We examined the link between four types of after-hours work: (i) long daily work hours (>10 hours); (ii) late evening work (after 21:00 hours); (iii) quick returns (<11 hours continued rest); and (iv) long weekly work hours (>40 hours a week) and employee health and wellbeing (ie, work–home conflict, detachment, burnout, and musculoskeletal pain), in fixed effects models. We stratified the analyses by working-time control.

**Results:**

The results support a link between late evening work, long daily and weekly work, and higher work–home conflict and lower detachment as well as between weekly work hours and higher burnout. The findings yielded limited support for work-time control as a moderating factor; the link between quick returns and burnout was only evident for employees with below-average work-time control.

**Conclusions:**

The four types of after-hours work were all independently related to at least one employee outcome, although the link with quick returns was only evident when work-time control was below average. The results are important for practitioners aiming to implement family-friendly and healthy practices.

Working from home, both during and outside regular work hours, has become increasingly accessible to a significant portion of the workforce. Approximately 40% of the EU working population is expected to be available for work outside working hours more than monthly (1). A growing field of research on working from home after regular work hours, examining technology-assisted supplementary work and availability after hours, finds negative consequences for employee health and wellbeing (2–4) also beyond what can be explained by the increase in total work hours (5, 6). Still, we lack essential knowledge on the severity and consequences of different types of after-hours work when work can be executed from home. To facilitate healthy work time practices, we investigate and differentiate between four types of after-hours work among employees with the opportunity to work from home: long daily and weekly work hours, quick returns and late evening work.

Prior research has demonstrated the negative health consequences of specific variations of working outside of “normal” work hours, including long daily (7–10) or weekly (11–13) work hours and quick returns (<1 hour continuous rest per day) (14–17). There is also some support for the negative effects of working evenings (18, 19). Consequently, several countries have implemented restrictions, such as the EU Working Time Directive, which dictates ≥11 hours continuous rest in every 24-hour period (20).

The current knowledge base is limited in several regards. First, specific types of after-hours work are often examined in isolation without accounting for other related work hours. Thus, the individual consequence of each type of work hour has not been examined. For example, studies on long daily work hours are predominantly done without accounting for weekly work hours (21). Consequently, it remains unclear whether working long daily hours is detrimental in itself or if the negative outcomes are primarily a result of employees also working extended weekly work hours. There is a need to differentiate between specific types of working after-hours and assess the unique outcome of each type while accounting for related types of after-hours work.

Second, there is a lack of studies examining the consequences of different types of after-hours work among employees who have the opportunity to work from home. For example, while a recent study showed that 21% of employees with the opportunity to work from home weekly report <11 hours continuous rest between workdays (22), studies examining quick returns have, until now, almost exclusively focused on shift workers. The consequences of specific types of after-hours work, such as quick returns, may be different when employees can execute their work from home. Consequently, there is a clear need to examine the consequences of specific types of working after-hours among employees with the opportunity to work from home.

The present study aims to examine the longitudinal link between four types of working after-hours and health and wellbeing outcomes among employees with the opportunity to work from home. We focus on four aspects of employee health and well-being linked to after-hours work in prior research: self-reported burnout (3), musculoskeletal pain (23–25), work–home conflict (2, 26), and psychological detachment (2, 3) (ie, disengaging mentally and not thinking about work during non-work hours (27)).

How the four types of after-hours work may affect employee health and well-being can be understood in terms of theories such as the effort–recovery model (28) and boundary theory (29). According to the effort–recovery model (28), high workload first leads to short-term responses, for instance mental or physical fatigue. This first reaction is reversible; when the workload ceases, employees will return to baseline levels through a recovery process. However, if the workload persists or recovery is insufficient, the reaction may evolve into a more long-lasting effect such as burnout or musculoskeletal pain. In all four types of after-hours work investigated in the current paper, recovery time is reduced or differently distributed throughout the day, potentially affecting the balance between effort and recovery. According to the boundary theory (29), employees create boundaries between different domains such as work and home. These boundaries can be flexible and permeable, such as when work can be enacted during non-work hours from home or while simultaneously being present in the home domain. When the boundaries become more flexible and permeable, each domain is more accessible while being present in another. Consequently, it is more difficult to detach from one domain while being present in another. Employees might also experience increased work–home conflict when working during non-work hours and experiencing expectations relating to work and home simultaneously. In turn, lack of detachment and work–home conflict may impair the recovery process, leading to health challenges such as increased burnout or musculoskeletal pain (27, 30).

In a fixed-effects design, analyzing variation within individuals, we expect: (i) Long daily work hours (>10 hours) are related to higher levels of burnout, work–home conflict, and musculoskeletal pain and lower levels of detachment from work, even after controlling for weekly work hours, quick returns and evening work [hypothesis 1 (H1)]; (ii) Evening work (after 21:00 hours) is related to higher levels of burnout, work–home conflict, and musculoskeletal pain and lower levels of detachment from work, even after controlling for long daily work hours, weekly work hours, and quick returns (H2); (iii) Quick returns (<11 hours continuous rest a day) are related to higher levels of burnout, work–home conflict, and musculoskeletal pain and lower levels of detachment from work, even after controlling for long daily work hours, weekly work hours, and evening work (H3); (iv) Long weekly work hours (>40 hours a week) are related to higher levels of burnout, work–home conflict, and musculoskeletal pain and lower levels of detachment from work, even after controlling for long daily work hours, quick returns and evening work (H4).

Moreover, the relationship between after-hours work and health and well-being is likely to differ among employees. Arguably, employees with high work-time control are more likely to have chosen to work after hours based on personal and family preferences, such as the need to adjust work hours to pick up children from school. Some findings have indeed supported that employees’ work-time control moderates the negative consequences of unhealthy work hour arrangements, such as the effect of shiftwork on mental health (31–34). Therefore, the present study aims to examine the role of work-time control for the relationship between working after-hours and employee health and wellbeing. We expect: (v) The relationship between each of the four types of after-hours work and employee health and wellbeing will be moderated by high work-time control, with a weaker relationship for employees with above-average work-time control (H5).

Two aspects of the Norwegian context are important for the examination of working time. First, each of the investigated types of after-hours work in the current study fall outside the standard working hours as defined by Norwegian labor regulations (35). Regulations state that normal working hours must not exceed 40 hours per 7 days, 10 hours per 24 hours, and, in line with EU regulations, an employee should have ≥11 hours continuous off-duty time per 24 hours (35). Working 21.00–06.00 hours is considered night work and not permitted unless necessitated by the nature of the work. The choice of work-hour types investigated are based on these regulations.

Second, the data was collected when a large part of the working force in Norway was under different injunctions due to the COVID-19 pandemic. During the first and third wave of the survey (February 2021 and January 2022), it was mandatory to work from home for all employees who could do so. During the second and fourth wave (August 2021 and September 2022) these injunctions were lifted. It is important to note that while the injunctions increased the proportion of employees who worked from home the whole day, working from home outside normal work hours was common in Norway also before the pandemic (1, 36).

## Method

### Sample and procedure

The sample was drawn from the Norlife Remote Work - Longitudinal Study (NorRemo-LS), a four-wave longitudinal questionnaire survey, following employees in February 2021, August 2021, January 2022 and September 2022, with approximately six-month intervals. The survey was distributed to 12 263 individuals from a Norwegian web panel (Kantar). Participation was based on informed consent. Individuals in the panel were predominantly recruited through probability-based representative samples utilizing double-opt-in procedure to ensure the representativeness of the sample. A total of 5038 employees responded at the first wave of the survey, resulting in a response rate of 41%. Additionally, 43 employees who did not participate in the first wave joined in subsequent waves. Of all respondents, 2041 (40%) employees participated in all four waves, 986 (19%) in three waves, 745 (15%) in two waves, and 1309 (26%) in one wave. In total, the responses yielded 13 921 observations.

**Figure 1:**
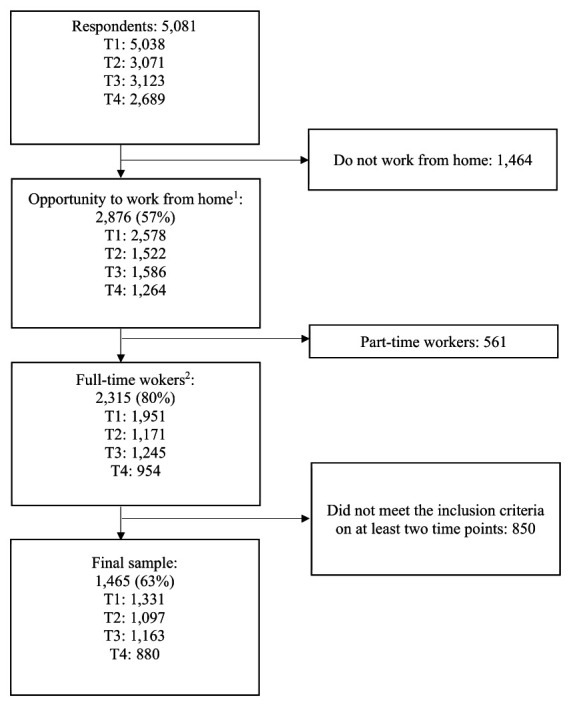
Flowchart of respondents and final sample. ^1^ Employees with the opportunity to work from home, and who work from home at least monthly either during the whole workday or outside normal work hours. ^2^ Contracted work hours 37–40 hours a week

The analysis sample was limited to employees who answered that they (i) had the opportunity to work from home, and (ii) work from home at least monthly either during the whole workday or outside normal work hours (see supplementary material, www.sjweh.fi/article/4198, Appendix A for questions). Furthermore, we limited the sample to employees with a fulltime position (ie, contracted work hours 37–40 hours per week) to reduce the risk of reversed causality since part-time employees might choose to work fewer weekly hours due to health challenges. Finally, we only included employees who had responded and met the inclusion criteria at more than one time point, resulting in a sample of 4421 observations from 1465 employees. For more details see figure 1.

### Variables

For full list of items see supplementary Appendix A.

### Work hour variables

***Long daily work hours*** was measured with the question: “During the past 6 months, how often have you worked >10 hours within a 24-hour period?”, ***late evening work:*** “During the past 6 months, how often have you worked after 21:00 hours?” and ***quick returns:*** “During the past 6 months, how often have you had <11 consecutive hours of complete free time during a 24-hour period? (eg, if you have finished a work session after 22:00 in the evening and started work by 09:00 in the morning the next day).” We focused on six months to correspond with the intervals between surveys. Each question had the response options “every day, several days a week, one day a week, monthly, less often, never” which for the purpose of this study was dichotomized into 1=weekly or more frequent, and 0=less than weekly.

***Weekly work hours*** were measured with the sum of two open-ended questions “How many hours per week do you usually work in total in your main employment relationship, including overtime hours and extra work at home in connection with this work?” Respondents with multiple jobs were also asked: “How many hours per week do you usually work in total in your other jobs, including overtime hours and extra work at home in connection with this work?” Because the focus of the present paper was on weekly work hours exceeding the legal limit, we operationalized long weekly work hours as a linear variable counting the number of work hours >40 hours. When employees worked ≤40 hours a week, they were given the value 0.

### Outcome variables

*Burnout* was measured with eight items from the exhaustion subscale from the Oldenburg Burnout Inventory (OLBI) (37) on a 4-point scale from 1=strongly disagree to 4=strongly agree. An example item included was: “There are days when I feel tired before I arrive at work”. Cronbach’s alpha was 0.88 in each wave.

*Musculoskeletal pain* was measured with 11 items based on the Standardized Nordic Questionnaires for the analysis of musculoskeletal symptoms (38). The respondents were asked “Have you at any time during the last 6 months had trouble (ache, pain, discomfort) in:” for each of the following body parts; neck, shoulder, upper back, elbows, lower back (lumbar spine), wrists/hands, hips, knee, ankles/feet. Response options were yes/no. The final variable was dichotomized as 1 for yes to at least one item, and 0 if no pain.

*Work–home conflict* was measured with four items from Gutek, Searle and Klepa (39), on a 5-point scale from 1=strongly disagree to 5=strongly agree. An example item includes: “On the job, I have so much work to do that it takes away from my personal interests”. Cronbach’s alpha ranged between 0.80 and 0.83 for the four waves.

*Detachment* was measured with four items from Sonnentag & Fritz (27) on a 5-point scale from 1=do not agree at all to 5=fully agree. An example item includes: “After work, I forget about work”. Cronbach’s alpha ranged between 0.88 and 0.89 for the four waves.

### Work-time control

Work-time control was measured using four items from Valcour's Control Over Work Time Scale (40) based on Thomas & Ganster (41). An example item is “How much control do you have over when you begin and end each workday”. Control was measured on a 5-point scale from 1=none to 5=a great deal. Cronbach’s alpha ranged between 0.83 and 0.85 for the four waves. To perform stratified analyses, we first standardized each employee’s level of work-time control as their mean across all waves. We standardized the level to avoid the results being influenced by control dropping when work hours increased. Secondly, we dichotomized work-time control into (i) below average and (ii) average and above level of work-time control based on the mean value (4.0) of work-time control in the sample.

### Statistical analyses

We analyzed the data using fixed effects panel regression in Stata using the xtreg fe command (StataCorp, College Station, TX, USA). Fixed effects utilize the longitudinal panel structure of the data by exclusively analyzing variations within each individual, ie, within-person variations. Specifically, for each employee, we analyzed how changes in each outcome correspond to changes in their work hours across waves. In contrast to cross-sectional analyses, no comparisons were made between employees in these models. Consequently, fixed effect models controlled for all stable (time-invariant) characteristics, such as gender, family situation, or stable personality traits, eliminating the need to adjust for these and similar control variables explicitly in the models. We used linear regression for all analyses. The coefficient for musculoskeletal pain, which was coded as a dichotomous outcome, was equivalent to average marginal effects (AME) in logistic regression (42).

The analyses were conducted in three steps. First, we conducted the analyses on the full sample of employees (N=1465). For each outcome, we ran two sets of analyses: (i) one separate for each type of after-hours work (long daily work hours, evening work, quick returns, and long weekly work hours) and (ii) one for all four types of after-hours work included simultaneously. Second, we conducted stratified analyses on employees with an above- and below-average level of work-time control. Third, we conducted supplementary analyses using random effects modeling (Stata xtreg re command) nesting responses within individuals, to test the statistical significance of a moderating effect of work-time control, as test comparing between groups are not possible in a fixed effects design. In the supplementary analyses, work-time control is also dichotomized to correspond with and test the results of the stratified analyses. In the second and third steps, all four types of after-hours work are included simultaneously.

## Results

The baseline characteristics of the employees included in the sample are presented in table 1. The sample is characterized by a high proportion of employees with higher education (ie, university and college degree). The total sample was relatively balanced on gender. Work-time control was relatively high in general; on a scale of 1–5, the average was 4.0. The largest industries were public administration, IT, and energy. The sample primarily consisted of office workers (eg, within the healthcare sector the most common professions in our sample are management and advisors). A correlation matrix of the key variables from the first wave of data (T1) is presented in table 2. All four types of after-hours work were closely related.

**Table 1:** Descriptive statistics of the sample. Note: Descriptive statistics for all employees in the sample from the first wave they are included in the sample. [SD-standard deviation.]

		N	%	Mean	SD
Total		1465	100		
Sex					
	Female	672	46		
Higher education					
	Yes	1081	74		
	Age			47	11
Lives with					
	Partner	508	35		
	Partner and child/children	560	38		
	Child/children	78	5		
	Alone	290	20		
	Other	25	2		
Sector					
	Central Government / Public Administration	328	22		
	Kindergarten / School / Education	71	5		
	Healthcare Services	64	4		
	Industry / Technology	87	6		
	Construction	78	5		
	Retail / Store	63	4		
	Culture / Sports / Organizations	63	4		
	Media / Advertising / PR / Information	51	4		
	Telecommunications / IT	132	9		
	Banking / Insurance / Finance	90	6		
	Business Services / Service Industry	57	4		
	Oil / Gas / Energy	112	8		
	Other	263	18		
Work from home for the whole workday					
	Every day	385	26		
	Several days per week	472	32		
	One day per week	197	13		
	Monthly	213	15		
	More seldom	183	12		
	Never	15	1		
Work from home outside normal work hours					
	Every day	90	7		
	Several days per week	545	37		
	One day per week	279	17		
	Monthly	259	20		
	More seldom	239	15		
	Never	53.0	4.4		
Extended days (>10 hour)					
	Multiple times a week	152	10		
	Once a week	194	13		
	Monthly	388	26		
	Less than monthly	477	33		
	Never	254	17		
Late evening (after 21:00 hours)					
	Multiple times a week	127	9		
	Once a week	131	9		
	Monthly	293	20		
	Less than monthly	516	35		
	Never	398	27		
Quick returns (<11 hours rest)					
	Multiple times a week	138	10		
	Once a week	81	6		
	Monthly	188	13		
	Less than monthly	463	33		
	Never	593	42		
Weekly work hours					
	Works >40 hour/week	490	33		
Hours worked >40 hours/week ^a^				5.4	3.8
Work hour control				4.0	0.6
Burnout				2.0	0.5
Work-home conflict				2.6	1.0
Detachment				3.0	1.0
Pain		1069	73		

^a^ Hours worked >40 hour/ week for employees who work >40 hours.

**Table 2:** Correlation Matrix (t1).

		1		2		3		4		5		6		7		8	
1	Long daily work hours (>10 hours)																
2	Evening work (after 21:00 hours)	0.47***															
3	Quick returns (<11 hours rest)	0.41***		0.47***													
4	Long weekly work hours (>40 hours)	0.48***		0.38***		0.29***											
5	Burnout	0.09***		0.07**		0.10***		0.07**									
6	Work-home conflict	0.32***		0.27***		0.23***		0.29***		0.68***							
7	Detachment	-0.29***		-0.27***		-0.19***		-0.31***		-0.21***		-0.36***					
8	Physical pain	-0.07**		-0.03		0.00		-0.06**		0.19***		0.12***		0.00			
9	Low work-time control	0.03		0.03		0.04		0.00		0.27***		0.25***		-0.11***		0.05*	

* P>0.05. ** P>0.01. *** P>0.001.

Longitudinal fixed effects analyses of the relationship between working time and the four employee outcomes (work–home conflict, detachment, burnout and musculoskeletal pain), H1–4 are presented in table 3.

**Table 3:** Fixed effects analyses of work hours and employee health and wellbeing. N (observations): 4564–5314. N (employees): 2105–2312

Working	Burnout			Work-home conflict			Detachment			Physical pain	
	M1 ^a^	M2 ^b^		M1 ^a^	M2 ^b^		M1 ^a^	M2 ^b^		M1 ^a^	M2 ^b^
Long daily work hours (>10 hours)	0.05 **	0.04		0.20 ***	0.12 ***		-0.22 ***	-0.17 ***		-0.00	-0.00
Evening work (after 21.00 hours)	0.01	-0.01		0.18 ***	0.10 *		-0.18 ***	-0.10 *		0.00	0.00
Quick returns (<11 hours rest)	0.04 *	0.02		0.12 ***	0.05		-0.12 ***	-0.06		-0.01	-0.01
Long weekly work hours (> 40 hours)	0.01 ***	0.01 ***		0.0 ***	0.03 ***		-0.03 ***	-0.02 ***		0.00	0.00

^a^ Each type of after-hours work is analysed separately (e.g., extended days and burnout, evening work and burnout etc.). ^b^ All types of after-hours work are included in the same analysis. * P>0.05. ** P>0.01. *** P>0.001.

H1 and H2 were partially supported. When employees worked long daily hours on a weekly basis, they experienced more burnout, work–home conflict and less detachment from work, compared to when they did not do so. The relationship between long daily work hours and work–home conflict and detachment remained significant when controlling for evening work, quick returns, and long weekly work hours (H1).

Similarly, when employees worked late evening on a weekly basis, they experienced more work–home conflict and less detachment from work, and the relationships remained significant after controlling for the three other types of after-hours work (H2).

H3 was also partially supported. When employees had quick returns on a weekly basis, they experienced higher levels of burnout and work–home conflict and less detachment, but none of these results remained significant after controlling for the three other types of after-hours work.

Finally, H4 was largely supported. Long weekly work hours were significantly related to burnout, work–home-conflict and detachment, after controlling for the three other types of after-hours work. Notably, no after-hours work type was associated with musculoskeletal pain.

The results stratified by below- and above-average level of work-time control are presented in table 4. The findings provide weak support for H5. Quick returns were significantly linked to higher burnout among employees with below-average level of work-time control, but not for employees with above-average work-time control.

**Table 4:** Fixed effects analyses of work hours and employee health and wellbeing for employees with below and above average level of control. For full radom effects analyses see Appendix B.

	Burnout				Work-home conflict				Detachment				Physical pain		
	Below ^a^	Above ^b^	RE ^c^		Below ^a^	Above ^b^	RE ^c^		Below ^a^	Above ^b^	RE ^c^		Below ^a^	Above ^b^	RE ^c^
Long daily work hours (>10 hours)	0.06	0.03			0.13 *	0.12 *			-0.17 **	-0.16 ***			0.01	-0.01	
Evening work (after 21.00)	-0.02	-0.01			0.09	0.11			-0.07	-0.12 *			0.05	-0.04	*
Quick returns (<11 hours rest)	0.07 *	-0.01	*		0.06	0.05			-0.06	-0.06			-0.05	0.01	
Long weekly work hours (>40 hour)	0.01 **	0.01 **			0.03 ***	0.03 ***			-0.02 *	-0.03 ***			0.01 *	-0.00	

^a^ Fixed effects. Sample of employees with below average level of work-time control. N (observations); 1857–2174. N (employees); 861–944. ^b^ Fixed effects. Sample of employees with average or above level of work-time control. N (observations); 2688–3082. N (employees); 1225–1328. ^c^ Random effect analyses. Performed on the full sample to test the interaction effect between work-time-control (low vs high) and the work hour types. * P>0.05. ** P>0.01. *** P>0.001.

Supplementary random effect analyses (Appendix B) show a significant interaction. Support for a moderating effect of work-time control beyond this was limited.

## Discussion

The current study investigated *whether* and *when* working outside normal work hours is detrimental to employee burnout, musculoskeletal pain, work–home conflict, and lack of detachment for employees with the opportunity to work from home. Our results partially support previous findings documenting the negative consequences of working from home outside regular work hours in general and also when controlling for weekly work hours (5, 6, 43, 44).

In particular, the results supported a relationship between working after-hours and higher work–home conflict and lower detachment. Indeed, long daily and weekly work hours, and evening work were all independently related to both work–home conflict and detachment, in line with former studies (2, 3, 26). The results are in line with boundary theory (29) suggesting that when the borders between work and home become more flexible and permeable, employees may experience more conflicting demands and find it harder to detach.

Notably, quick returns were no longer significantly related to work–home conflict or detachment after controlling for the three other work hour variables. The results indicate that for employees who can work from home, a relationship to work–home conflict and detachment is primarily caused by employees also working more hours in total, more daily hours and more frequently during the evening.

The results only provided partial support for a relationship between after-hours work and burnout. Long weekly work hours were independently related to burnout. Furthermore, quick returns were also independently related to burnout, but only for employees with a below-average level of work-time control. The negative health effects of quick returns are well documented (14–17, 45), but studies examining quick returns have, until now, almost exclusively focused on shift workers. Quick returns while working from home are clearly different from quick returns in shift work. Our results support that, among employees with the opportunity to work from home, quick returns are only related to burnout if not accompanied by a high level of work-time control.

In contrast to former studies (23–25), we did not find a link between any of the after-hours work types and musculoskeletal pain. One potential reason may be due to reversed causality. In the cross-sectional correlation matrix, long daily and weekly work hours are related to less musculoskeletal pain, potentially caused by employees with more pain working fewer hours. If this is the case, it might mask a negative impact of working hours on pain in the longitudinal analyses.

Finally, apart from the relationship between quick returns and burnout, we found limited support for a moderating role of work-time control. Prior studies have supported that work-time control may moderate the consequences of unhealthy work hours (31–34). The opportunity to work from home after hours and the generally high level of worktime control in the current sample might also reflect a highly individualized responsibility for employees to manage work tasks within work hours and refrain from overwork.

### Practical implications

Flexible work arrangements allowing employees to execute part of their work outside the workplace and regular workhours have received attention as family-friendly policies (46–48). The actual benefits of such policies for the employees have, however, been questioned as studies indicate that this flexibility might also increase total work hours (49, 50). Our results showing that multiple types of after-hours work are independently related to higher work–home conflict contribute to these concerns. Practitioners hoping to facilitate family-friendly policies with flexible work arrangements should make sure to counteract potential overwork among employees.

### Strengths and limitations

The current study utilizes a large longitudinal data set spanning four waves over two years. By comparing each employee to themself, we can exclude all time-invariant differences between employees, such as stable personality traits and demographic variables. By simultaneously examining four different types of after-hours work, we investigated the unique relationship between each type and employee health and well-being outcomes. Still, caution must be taken when interpreting the causality of these findings as we cannot exclude alternative causal pathways. For instance, it may be possible that employees are more likely to work late evenings when they struggle to detach and not vice versa.

Common method bias and social desirability in answers are well-known limitations in self-reported questionnaire surveys. Our study utilizes the variation within each individual’s responses, which removes any time-constant individual patterns in answering surveys, such as high level in general agreeableness or social desirability, while only analyzing the variation between time points. Still, future research could benefit from investigating the same questions using objective measures of after-hours work patterns.

It is important to note that the absence of evidence is not evidence of absence (51). While the current data have a relatively large sample, the risk of type 2 error may still be present. For instance, the dichotomization of three of the after-hours work measures might have increased the possibility of wrongfully finding non-significant results as the variance is reduced. Other thresholds for dichotomization might have provided different results. Further studies should investigate the after-hours work types also with different thresholds.

Three of the four waves were collected during the COVID pandemic. The pandemic might have influenced the findings by increasing (or decreasing) various types of working after hours. However, as working from home outside normal hours was common in Norway before the pandemic and still is after, it is likely that the impact of COVID on our findings were minor. The data also showed minor differences in which industries had the opportunity to work from home at T1 (when working from home was mandated) and T4 (when all restrictions were lifted).

### Concluding remarks

Working from home after regular hours has become a common occurrence, and a growing field of research is supporting negative health and well-being consequences as a result. The current study is among the first to investigate the longitudinal link between four different types of after-hours work and employee health and well-being among employees with the opportunity to work from home. Our results suggest that working long daily work hours and late evenings is linked to reduced detachment from work and increased work–home conflict beyond what can be explained by related types of after-hours work. Furthermore, our results suggest that quick returns are related to increased burnout, but only among employees with below-average work-time control. Practitioners aiming to implement family-friendly and healthy work-hour practices using flexible work arrangements should work to avoid inadvertently increasing work–home conflict and deteriorating recovery through more after-hours work.

## Supplementary material

Supplementary material
