# Supplementary material for: The consequences of after-hours work: a fixed-effect study of burnout, pain, detachment and work–home conflict among Norwegian workers
Source: Scand J Work Environ Health. 2024 Dec 30;51(1):38–47. doi: 10.5271/sjweh.4198 (PMC11710905; doi:10.5271/sjweh.4198)
Supplement: Supplementary material [file SJWEH-51-38-S001.pdf]

The consequences of after-hours work: a fixed-effect study of burnout, pain, detachment and work–home conflict among Norwegian workers[1]

by Vilde Hoff Bernstrøm, PhD,[2] Mari Ingelsrud, PhD, Wendy Nilsen, PhD

1. Supplementary material
2. Correspondence to: Vilde Hoff Bernstrøm, Work Research Institute, OsloMet – Oslo Metropolitan University, Oslo, Norway. [E-Mail: vilde.bernstrom@oslomet.no]

Appendix A: Survey items

Possibility to work from home
Do you have the opportunity to work from home if you wish to? (By home we mean home and other places you can choose)
Response format: Yes, as a regular arrangement; Yes, but only when needed; No; I don't know

Frequency of work from home
How often do you work from home the whole day?
How often do you work from home outside normal work hours?
Response format: Every day, several days a week, one day a week, monthly, less often, never

Long daily work hours
During the past 6 months, how often have you worked more than 10 hours in a 24-hour period?
Response format: Every day, several days a week, one day a week, monthly, less often, never

Evening work
During the past 6 months, how often have you worked after 9 p.m.?
Response format: Every day, several days a week, one day a week, monthly, less often, never

Quick returns

During the past 6 months, how often have you had less than 11 consecutive hours of complete free time during a 24-hour period? (e.g., if you have finished a work session after 22:00 in the evening and started work by 9:00 in the morning the next day).

Response format: Every day, several days a week, one day a week, monthly, less often, never

Long weekly work hours

How many hours per week do you usually work in total in your main employment relationship? Including overtime hours and extra work at home in connection with this work

Response format: open

You answered that you have more than one job/employer. How many hours per week do you usually work together in your other jobs/working relationships? Including overtime hours and extra work at home in connection with this work.

Response format: open

Control Over Work Time Valcour (2007) Thomas and Ganster (1995)

How much control do you have over…

… when you begin and end each workday

… the number of hours you work each week

… when you can take a few hours off

… when you take vacations or days off

Response format: 1.” None” to 5. “A great deal”

Burnout - Exhaustion (Demerouti, Mostert, & Bakker, 2010)

There are days when I feel tired before I arrive at work

After work, I tend to need more time than in the past in order to relax and feel better

I can tolerate the pressure of my work very well

During my work, I often feel emotionally drained

After working, I have enough energy for my leisure activities

After my work, I usually feel worn out and weary

Usually, I can manage the amount of my work well

When I work, I usually feel energized
Response format: 1. “Strongly agree”, 2. “Agree”, 3. “Disagree”, 4. “Strongly disagree”

Work-family conflict (Gutek, Searle, & Klepa, 1991)
After work, I come home too tired to do some of the things I'd like to do.
On the job I have so much work to do that it takes away from my personal interests.
My family/friends dislike how often I am preoccupied with my work while I am at home.
My work takes up time that I'd like to spend with family/friends.
Response format: 1. “Strongly disagree” to 5. “Strongly agree”

Detachment (Sonnentag & Fritz, 2007)
After a workday…
… I forget about work
… I don’t think about work at all
… I distance myself from my work
… I get a break from the demands of work
Response format: 1. “Do not agree at all” to 5. “Fully agree”

Musculoskeletal pains. Simplified from (Kuorinka et al., 1987)
Have you at any time during the last 6 months had trouble (ache, pain, discomfort) in;

- Neck
- Shoulder
- Upper back
- Elbows
- Lower back (lumbar spine)
- Wrists/Hands
- Hips
- Knee
- Ankles/Feet

Response format: 1. Yes 0. No

Appendix B

Appendix B: Random effects analyses of work hours, work-time control and employee health and wellbeing

	Burnout	Work-home conflict	Detachement	Physical pain
Long daily work hours (>10 hours)	0.0600**	0.398***	-0.372***	-0.0568**
Low control	0.275***	0.457***	-0.204***	0.0310
Interaction	0.0428	0.0748	0.000212	0.0524
Evening work (after 21.00)	0.0395	0.392***	-0.422***	-0.0534*
Low control	0.285***	0.482***	-0.223***	0.0302
Interaction	0.0103	-0.0234	0.0992	0.0706*
Quick returns (<11 hours rest)	0.0255	0.277***	-0.245***	-0.0135
Low control	0.273***	0.481***	-0.221***	0.0434*
Interaction	0.0773*	0.00992	0.0451	-0.000483
Long weekly work hours (> 40 hour)	0.00874**	0.0534***	-0.0602***	-0.00675**
Low control	0.279***	0.471***	-0.237***	0.0308
Interaction	0.00470	0.00948	0.0104	0.00635
